# Kinase analysis of penile squamous cell carcinoma on multiple platforms to identify potential therapeutic targets

**DOI:** 10.18632/oncotarget.15558

**Published:** 2017-02-21

**Authors:** Eddy S. Yang, Christopher D. Willey, Amitkumar Mehta, Michael R. Crowley, David K. Crossman, Dongquan Chen, Joshua C. Anderson, Gurudatta Naik, Deborah L. Della Manna, Tiffiny S. Cooper, Guru Sonpavde

**Affiliations:** ^1^ Department of Radiation Oncology, University of Alabama, Birmingham (UAB), Birmingham, Alabama, USA; ^2^ Department of Medicine, Section of Oncology, UAB School of Medicine, Birmingham, Alabama, USA; ^3^ Department of Genetics, UAB School of Medicine, Birmingham, Alabama, USA; ^4^ Department of Medicine, Division of Preventive Medicine, Biostatistics and Bioinformatics Shared Facility, UAB Comprehensive Cancer Center, Birmingham, Alabama, USA

**Keywords:** penile squamous cell carcinoma, kinases, DNA, RNA, protein

## Abstract

Penile squamous cell carcinoma (PSCC) is an orphan malignancy with poorly understood biology and suboptimal systemic therapy. Given that kinases may be drivers and readily actionable, we performed comprehensive multiplatform analysis of kinases in PSCC tumor and normal tissue. Fresh frozen tumors were collected from 11 patients with PSCC. After macrodissection to demarcate tumor from normal tissue, the samples underwent multiplatform analysis of kinases. Next Generation Sequencing (NGS) of 517 kinase genes was performed using Agilent Kinome capture and run on the Illumina MiSeq at PE150bp. The NanoString nCounter® platform analyzed the expression of 519 kinase genes. Kinase activity of tissue lysates was measured using PamStation®12 high-content phospho-peptide substrate microarray system. Network mapping was done with GeneGo MetaCore™ and upstream kinase prediction was performed with BioNavigator and the Kinexus database. Ingenuity pathway analysis was performed to integrate elevated kinase activity and gene over-expression with coexisting missense mutations at DNA level. Top pathways upregulated in both the kinase activity and gene expression platforms were PTEN, STAT3, GNRH, IL-8 and B cell receptor signaling. Potentially relevant missense mutations were seen in 176 kinase genes, with the top altered pathways overlapping with gene overexpression being GNRH, NF-kB and STAT3 signaling. ERBB2, ERBB3 and SYK were altered on NGS and also exhibited elevated kinase activity. To summarize, multiplatform comprehensive analysis of kinases discovered potential drivers of PSCC and actionable therapeutic targets. Translational studies are necessary to validate the functional relevance of our data to make advances in this rare malignancy.

## INTRODUCTION

The biology and drivers of penile squamous cell carcinoma (PSCC), an orphan disease, are poorly understood. Systemic therapy for metastatic PSCC yields poor outcomes, with a median overall survival (OS) of 6 to 9 months [1–10]. Second-line chemotherapy with taxanes is marginally active with a median survival of ∼6 months [11]. Epidermal growth factor receptor (EGFR) inhibitors and vascular endothelial growth factor (VEGF) inhibitors have exhibited signals of modest activity [12–15]. Hence, substantial advances in systemic therapy are likely to emerge only if trials are informed by improved knowledge of biology.

A systematic and extensive genomic analysis of PSCC has not taken place. Indeed, the Cancer Genome Atlas (TCGA) has not selected PSCC for investigation. In this context, a targeted in-depth molecular analysis of kinases may provide valuable information to guide drug development. Multiple kinase inhibitors are already approved to treat a range of malignancies. Kinases are common distal drivers of disease and are readily actionable. We hypothesized that consistently altered kinases and pathways in tumor tissue identified through integrating multiple platforms spanning DNA, RNA and protein level data may provide excellent insights regarding drivers of disease and therapeutic targets. Hence, we performed comprehensive multiplatform analysis of kinases in PSCC tumor tissue and adjacent normal tissue to discover potentially actionable therapeutic targets.

## RESULTS

### Tissue sample characteristics

Fresh frozen PSCC tumor tissue was available from 11 patients with PSCC. The median age was 58 years (range 45-70). Two patients were African-American and the rest were Caucasian. Following histologic macrodissection, adequate adjacent normal tissue was available from 3 of these 11 patients for NGS and NanoString, and from 4 patients for the kinase activity assay. The tumor tissue samples had a median cellularity of 100% (range 50-100%), the median percentage of tumor cells with nuclei was 80% (range 30-90%) and the median proportion of necrosis was 0% (range 0-30%).

### Multi-platform kinase analysis of tumor and normal tissue

**Figure 1:**
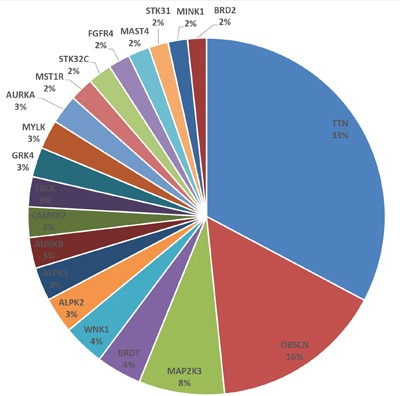
Genes with missense mutations The pie chart shows kinase genes with the most number of missense mutations (with the denominator being the total number of missense mutations in all kinase genes).

Missense mutations were observed in 176 kinase genes overall in tumor tissue samples compared to adjacent normal tissue. The top 10 mutated genes overall when comparing the 11 tumors vs. 3 normal tissue samples regardless of specific mutations or number of mutations per gene were OBSCN, TTN, CAMK2B, RPS6KA4, FES, PAK4, WNK1, STK25, CAMK2G and TNK2 (Supplementary Table 3). When examining by number of missense mutations per gene, with the denominator being the total number of missense mutations in all genes, TTN, OBSCN and MAP2K3 were the top genes (Figure 1). Key mutated genes in addition to top 10 genes above were PAK4, TNK2, ERBB2 and ROR2. Copy number analysis based on exome sequencing data using VarScan software showed that the genome area hosting ATM, BRCA2 and CCND2 genes had >2-fold intensity changes in all three paired tumor samples compared to matched normal tissue samples [16, 17].

**Figure 2:**
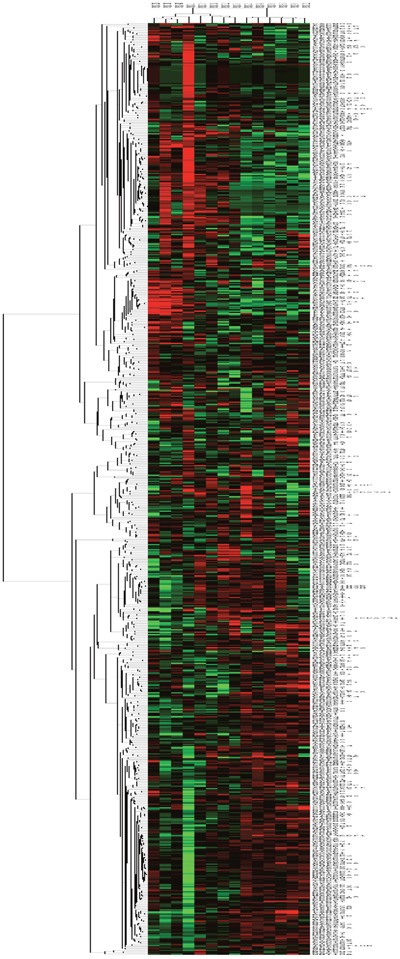
Kinase gene expression (NanoString) of normal and tumor samples Gene expression heatmap showing unsupervised hierarchical kinase gene expression of the 3 normal sample columns on the left and 11 tumor samples on the right, with red showing overexpressed and green representing down-regulated gene expression; the heatmap shows the broad differences in kinase gene expression between normal and malignant tissue.

The NanoString gene expression analysis showed substantial differences between tumor and normal samples (Figure 2). The top 10 over-expressed genes in the tumor samples compared to normal samples were: PLK1, CDK6, GSG2, BUB1, BUB1B, CASK, LIMK1, AURKB, CHEK1 and PBK (Supplementary Table 4). Paired tissue analysis in 3 evaluable patients also identified PLK1, BUB1, and PBK as the top genes overexpressed in tumor relative to adjacent normal samples.

**Figure 3:**
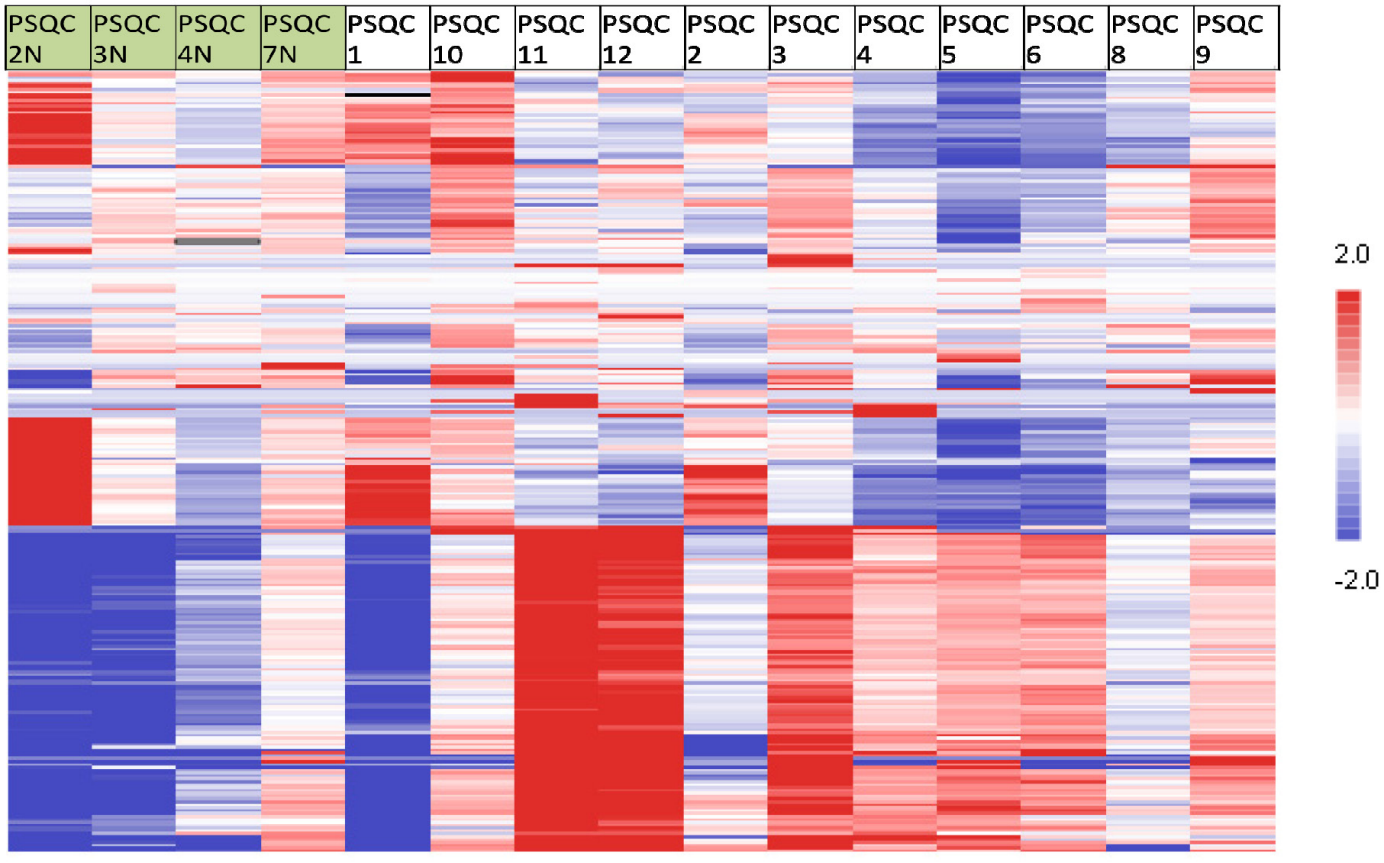
Kinase activity (Pamgene) of tumor and normal samples Heatmap showing kinase activity in 11 tumors (on right) and 4 normal samples (on left) with red showing relative high activity and blue representing relative lower activity; the heatmap shows the broad differences in kinase activity between normal and malignant tissue.

The kinase activity analysis showed substantial differences in kinase activity between tumor and normal samples with kinomic signatures clustered by peptide in Figure 3. The top 10 unpaired normal (n=4 samples) to tumor (n=11 samples) increased protein kinases were SYK, ZAP70, ARG (ABL2), ERBB3, ABL, TEC, MER (MERTK), AXL, EGFR and ERBB2 (Supplementary Table 5). Paired tissue analysis in 3 patients identified ARG, AXL, TYRO3 and ZAP70 as increased in tumor relative to adjacent normal.

### Integration of kinase data from DNA, RNA and protein activity levels

**Figure 4:**
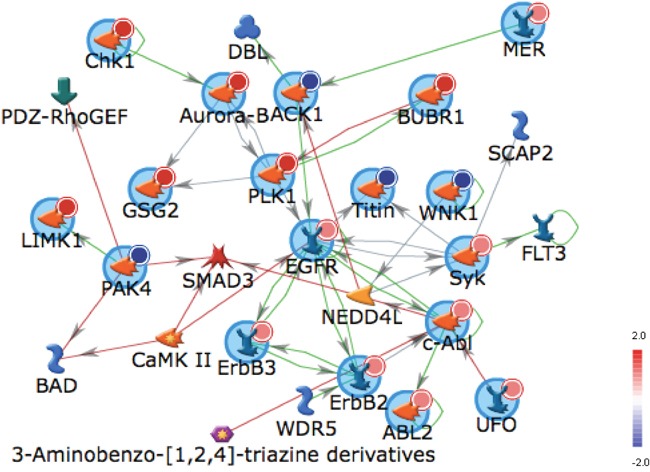
Network Mapping of Combined Molecular Profiling of PSCC Auto Expand EGFR-centric Network modeling (max 50 nodes, canonical deselected) of overexpressed genes (dark red), mutated genes (blue) and enzymatically activated proteins (light red).

Top pathways upregulated in tumors when integrating both the kinase activity and gene expression platforms were GNRH, PTEN, STAT3, IL-8 and B cell receptor signaling. The top upregulated pathways in tumors after integrating data from kinase missense mutations on exome sequencing and gene expression were GNRH, NF-kB, STAT3 and Nitric Oxide (NO) and Reactive Oxygen Species (ROS) in macrophages pathways. ERBB2, ERBB3 and SYK were functionally more active overall in tumors and TYRO3 was more active in paired tumor samples and also mutated on kinase gene sequencing. Platform specific alterations were combined including the most overexpressed genes, mutated genes, and unpaired, or paired increased activity kinases and uploaded via Uniprot ID to MetaCore™ (portal.genego.com) for biological network mapping via literature curated and annotated interactions that led to generation of the EGFR centric network (Figure 4).

## DISCUSSION

Multiplatform comprehensive PSCC tumor tissue analysis of kinases at the DNA, RNA and protein activity levels discovered several potential drivers of PSCC and actionable therapeutic targets. When examining consistently altered pathways or kinases across more than one platform, the GNRH, PTEN, STAT3, IL-8, B cell receptor, NF-kB and NO-ROS in macrophages pathways as well as the kinases ERBB2, ERBB3 and SYK were upregulated in tumor tissue. The GnRH and STAT3 pathways were upregulated across all 3 platforms.

Two genes coding for giant proteins and known to be involved in muscle homeostasis and more recently associated with multiple malignancies, OBSCN (Obscurin) and TTN (Titin) were the top kinase genes with missense mutations [18]. OBSCN is expressed in striated muscle and plays a role in myofibrillogenesis and cytoskeletal homeostasis. Recent data indicate OBSCN mutations in multiple malignancies, suggesting a potential cancer-promoting role [19]. Loss of OBSCN promoted breast epithelial cell survival, epithelial mesenchymal transition and metastasis [20]. TTN mutations have recently been reported in triple negative breast cancer and colon cancer, although therapeutic implications are unknown [21]. However, the relation of these genes with HPV and their therapeutic actionability is unknown. Genes previously implicated in prostate cancer (PAK4, TNK2) and genital dysplasia (ROR2) also demonstrated mutations.

While this study is limited by the small number of tumor and normal tissue samples, the strengths are the excellent quality of fresh frozen PSCC with excellent tumor cellularity and presence of nuclei coupled with low levels of necrosis. The normal tissue was selected based on adjacent histologically normal epithelium, which may also harbor kinase alterations due to a field effect. Nevertheless, given that we aimed to identify major kinases driving malignant transformation, our methodology can be justified to identify major potentially cancer-promoting kinase alterations. The rarity of this disease compounded by poor funding renders studies of a large number of tumors particularly challenging. A formal comparison of PSCC kinase alterations with kinase alterations in squamous cell carcinomas arising from other organs and partly driven by HPV (e.g. anus, cervix, head and neck) may be considered to identify similarities and for serving as potential surrogates in the absence of large PSCC datasets. However, the caveat is that identical molecular alterations harbored in different malignancies may have different context-driven therapeutic implications. Moreover, the in depth of analysis on 3 different platforms provides robustness to our results. This study focused on kinases as kinases are highly actionable with multiple kinase inhibitors available or being developed for multiple malignancies and new kinase inhibitors are generally more easily designed relative to other classes of agents.

A few recent studies have preliminarily reported somatic gene alterations in PSCC. Three other separate studies with modest sample size (tumors from 11- 43 patients) reported gene alterations in EGFR, PIK3CA, NOTCH1, CDKN2A, CCND1, AR, JAK2, JAK3, ALK, PTEN and BRCA2 [22–24]. These previous reports appear partly concordant with our findings suggesting the potential importance of signaling in the EGFR/HER family, the PI3K pathway, androgen pathway signaling via AR, JAK-STAT signaling, and deficits in DNA damage repair due to BRCA alterations. It is noteworthy that our study focused exclusively on kinases in contrast with these prior studies, which evaluated a different panel of cancer associated genes. Therefore, inhibitors of components of these pathways may yield clinical benefits, and such agents are already commercially approved for other malignancies. The possible value of inhibiting androgen signaling is particularly intriguing, given that androgens play a major role in penile structure and function. Androgen synthesis inhibitors (GnRH agonists/antagonists, abiraterone acetate) and AR inhibitors (enzalutamide, bicalutamide) play a major role in treating prostate cancer, another androgen driven disease, and possibly may benefit patients with PSCC. Conversely, the overexpression of IL-8 signaling, which promotes angiogenesis, may suggest relative resistance to VEGF inhibitors, as shown in other malignancies. The relatively large number of mutations in kinase genes is also noteworthy, which suggests a possibly large neoantigen burden translating to activity of T-cell checkpoint inhibitors, such as Programmed Death (PD)-1 and cytotoxic T-lymphocyte antigen (CTLA)-4 inhibitors. Intriguingly, the presence of mutations coupled with increased activity of TYRO3, a receptor tyrosine kinase, involved in inhibition of immune response mediated by toll-like receptors, supports the possible relevance of immunotherapy. Clearly, these hypothesis-generating findings from our exploratory study warrant external validation in larger datasets coupled with functional validation in further studies.

Unfortunately, preclinical PSCC models and cell-lines to investigate therapeutic approaches have not been well established. Thus, preclinical validation of the functional importance of molecular alterations is challenging. Hence, clinical trials with an objective of seeking signals of biologic and anti-tumor activity when using inhibitors of these pathways require attention. In this context, it is known that cisplatin-based chemotherapy appears to be the most optimal first-line regimen, which accords with the presence of defects in DNA damage repair engendered by BRCA alterations [2]. Additionally PARP inhibitors may warrant evaluation in those with somatic BRCA alterations. Similarly, EGFR inhibitors have already demonstrated signals of activity in anecdotal reports [13]. Indeed, ongoing phase II trials are evaluating HER family inhibitors, dacomitinib (NCT01728233) or afatinib (NCT02541903), as neoadjuvant therapy and salvage therapy, respectively. Another ongoing phase II trial (NCT02837042) is investigating the efficacy and safety of pembrolizumab, a PD-1 inhibitor, as salvage therapy.

To summarize, this is the first study to our knowledge to evaluate kinases on DNA, RNA and protein activity platforms and offer insights regarding a number of potentially actionable therapeutic targets to treat PSCC. A single dominant therapeutic target appears elusive and the disease seems to be characterized by substantial molecular heterogeneity. The combination of rarity of this disease coupled with molecular heterogeneity may render drug development particularly challenging. Nevertheless, a concerted international effort to develop therapy in a trial bearing an umbrella design to select patients for therapy based on molecular profile should be considered.

## MATERIALS AND METHODS

### Tissue samples

Fresh frozen PSCC tissue samples from the primary penile tumor (T) with adjacent normal tissue (N), were provided by the Cooperative Human Tissue Network (CHTN) based at UAB. CHTN complies with federal human subjects regulations (The “Common Rule;” 45 CFR part 46) to collect and distribute biospecimens. Patients were previously untreated by systemic therapy or locoregional radiotherapy. Surgically resected tumor samples were obtained from patients and snap frozen with or without O.C.T.^™^ in liquid nitrogen (LN02) and stored in plastic cassettes in LN02 tanks [25]. All specimens had independent central pathological assessment for diagnosis confirmation prior to release to the investigator. The tissue underwent histologic macrodissection to demarcate tumor from normal tissue. An IRB approved protocol (X120917005) at The University of Alabama at Birmingham (UAB) permitted the study.

### Harvesting of DNA, RNA and protein lysate from tissue

Based on demarcation by the pathologist, adequate tumor and normal tissue were isolated to provide genomic double stranded DNA, RNA and protein lysate. Genomic DNA was isolated using phenol-chloroform extraction by standard molecular biology techniques. dsDNA was quantified using the Qubit Broad Range dsDNA kit (Invitrogen, Carlsbad, CA) and DNA quality assessment was performed by gel electrophoresis. RNA was harvested using the Qiagen RNeasy kit and quality of RNA was confirmed via the 260/280 ratio using NanoDrop. For protein kinase activity profiling, tissue lysed in M-PER lysis buffer (ThermoFisher Scientific, Waltham, MA) containing 1:100 Halts’ protease and phosphatase inhibitors (ThermoFisher) was used. Protein was quantified by the BCA protein determination method (ThermoFisher).

### Tissue molecular analyses on DNA, RNA and protein kinase activity platforms

The samples underwent analysis of kinases using different platforms to assess DNA, RNA and kinase activity. Next Generation Sequencing (NGS) for 517 kinase genes (Supplementary Table 1) was performed using the Agilent Kinome capture and run on the Illumina MiSeq at PE150bp. This MiSeq sequencer allows targeted gene sequencing to enable 3 separate runs generating an average of 11.35 million reads per run yielding an average 3.46 Gb of sequence data.

Gene Expression profiling for 519 kinase genes and 8 housekeeping genes (Supplementary Table 2) was performed using the NanoString nCounter® analysis system [26, 27]. RNA (≥100 ng) without the need for amplification was input and hybridized for 19 hours at 65°C in the nCounter® NanoString platform, which utilizes digital counting employing two ∼50 hybridizing base probes per RNA. A codeset specific to a 100-base region of the target mRNA was custom designed by NanoString Technologies using a 3′ biotinylated capture probe and a 5′ reporter probe tagged with a specific fluorescent barcode, creating two sequence-specific probes for each target transcript. Automated removal of excess probe was performed by immobilization of probe-transcript complexes on a streptavidin-coated cartridge. Background hybridization was determined using spiked-in negative controls. All signals below mean background plus 2 standard deviations were considered to be below the limits of detection, and set to mean background. A normalization factor was calculated from the spiked-in exogenous positive controls in each sample and applied to the raw counts from the nCounter™ output data.

Global kinase activity of tissue lysates was measured in the UAB Kinome Core using PamStation®12 high-content phospho-peptide substrate microarray system (PamGene International,‘s-Hertogenbosch, Netherlands) [28–30]. A total of 2-10 μg of soluble protein lysates were loaded onto the appropriate PamChip® [PTK (tyrosine kinome) or STK (serine/threonine kinome)] in kinase buffer. This platform utilizes a high throughput peptide microarray system analyzing 144 individual tyrosine phosphorylatable peptides, or 144 serine and threonine phosphorylatable peptides imprinted and immobilized in a 3D format to assess kinomic activity in lysates. FITC conjugated phospho-specific antibodies (PamGene) are used for visualization during and after lysates are pumped through the array. The peptide phosphorylation signal is captured via a computer-controlled digital (CCD) camera. Peptide spot intensity was captured across multiple exposure times (10, 20, 50, 100, 200 ms) and the slope was taken, multiplied by 100, and log2 transformed. These values were used for comparative analysis.

### Bioinformatics analyses

Tumor tissue was compared with normal tissue for all platforms. For analysis of kinase exome sequencing, raw sequence data in FASTQ format were aligned to human reference genome, quantified, and compared by using a local instance of Galaxy (galaxy.uabgrid.uab.edu). Digital raw counts of RNA abundance by NanoString were normalized using internal controls and housekeeping genes. Pathway analysis was performed using Ingenuity Pathway Analysis (IPA) software to integrate over-expression at the activity and gene expression level with coexisting missense mutations at DNA level. To analyze kinase activity on the PamGene platform, advanced network modeling of altered phosphopeptides was performed using MetaCore™ (Thompson Reuters) [28]. The peptides were analyzed for upstream kinase prediction using the Kinexus Kinase Predictor (www.phosphonet.ca) with scoring according to the % hits (occurrence divided by the number of residues with kinase information) of a kinase within the top 10 list for each peptide as previously reported [31]. Kinases identified as increased via multi-platform profiling, were uploaded to MetaCore™ as Uniprot ID's and an Auto Expand Network modeling (max node <50, canonical deselected, orphan nodes hidden, compounds removed) was used to generate a network model.

## RELEVANT DISCLOSURES

- Eddy S. Yang: Consultant for Nanostring technologies
- Guru Sonpavde: Consultant for Bayer, Sanofi, Pfizer, Novartis, Eisai, Janssen, Amgen, Astrazeneca, Merck, Genentech, Argos, Agensys, BMS, Exelixis; Research support to institution from Bayer, Onyx, Celgene, Boehringer-Ingelheim, Merck, Pfizer, Sanofi; Author for Uptodate; Speaker for Clinical Care Options
- Christopher D. Willey: Consultant for Varian Medical Systems

## SUPPLEMENTARY MATERIALS TABLES










