# Supplementary material for: Kinase analysis of penile squamous cell carcinoma on multiple platforms to identify potential therapeutic targets
Source: Oncotarget. 2017 Feb 21;8(13):21710–8. doi: 10.18632/oncotarget.15558 (PMC5400617; doi:10.18632/oncotarget.15558)
Supplement: Supplementary file 1 [file oncotarget-08-21710-s001.pdf]

Kinase analysis of penile squamous cell carcinoma on multiple platforms to identify potential therapeutic targets

SUPPLEMENTARY TABLES

Supplementary Table 1: Kinase genes that underwent Next Generation Sequencing

See Supplementary File 1

Supplementary Table 2: Kinase genes evaluated for expression (RNA) by NanoString

See Supplementary File 2

Supplementary Table 3: Genes identified to have missense mutations

See Supplementary File 3

Supplementary Table 4: Raw data for gene expression in all tumor and normal samples using NanoString

See Supplementary File 4

Supplementary Table 5: Kinase activity changes in tumor compared to normal tissue (PamChip) in unpaired (A) and paired (B) samples

A.

Kinase Increased in PSCC	uniprot	hits	%hits
SYK	P43405	4.5	75
ZAP70	P43403	3.5	58.33333333
ARG (ABL2)	P42684	3.5	58.33333333
ERBB3	P21860	3	50
ABL	P00519	3	50
TEC	P42680	2.5	41.66666667
MER (MERTK)	Q12866	2	33.33333333
AXL	P30530	2	33.33333333
EGFR	P00533	2	33.33333333
ERBB2	P04626	2	33.33333333

B.

Kinase Increased in PSQCC (paired)	uniprot	hits	%hits
ARG (ABL2)	P42684	11	36.66667
AXL	P30530	10	33.33333
TYRO3	Q06418	9.5	31.66667
ZAP70	P43403	9	30
BMX	P51813	8.5	28.33333
FRK	P42685	8.5	28.33333
TEC	P42680	8	26.66667
MER (MERTK)	Q12866	8	26.66667
ERBB3	P21860	7.5	25
SYK	P43405	7.5	25
ABL	P00519	7.5	25
EGFR	P00533	7.5	25
ERBB2	P04626	7.5	25
